# Dialysis Access-Associated Steal Syndrome in High-Risk Patients Undergoing Surgery for Hemodialysis Access: A Systematic Review and Meta-Analysis of Preventive Operative Techniques

**DOI:** 10.7759/cureus.49612

**Published:** 2023-11-28

**Authors:** Saeed S Alqahtani, Fahad K Aljaber, Bader Y Alsuwailem, Yazeed A AlMashouq, Bander G AlHarbi, AlSayed M Elawad

**Affiliations:** 1 Vascular Surgery, Charité-Universitätsmedizin Berlin, Berlin, DEU; 2 Vascular Surgery, Prince Sultan Military Medical City, Riyadh, SAU; 3 Vascular Surgery, King Fahad Medical City, Riyadh, SAU; 4 Vascular Surgery, Samsung Medical Center, Seoul, KOR

**Keywords:** systematic review, arteriovenous graft, arteriovenous fistula, steal syndrome, end-stage renal disease, hemodialysis

## Abstract

This systematic review and meta-analysis examine preventive operative techniques in high-risk patients undergoing surgery for hemodialysis access to mitigate the risk of Dialysis Access-Associated Steal Syndrome (DASS). Chronic kidney disease often leads to end-stage renal disease (ESRD), necessitating dialysis. Successful vascular access is crucial for efficient dialysis, but complications, such as DASS, pose significant challenges. DASS redirects arterial blood flow, affecting populations undergoing arteriovenous access surgery. This study aims to assess preventive strategies, including distal revascularization with interval ligation (DRIL) and extension techniques. A systematic search of PubMed, Cochrane Library, EMBASE, and Web of Science until 2022 identified 11 relevant studies. The inclusion criteria comprised non-pediatric hemodialysis patients reporting outcomes related to patency and complications. The data were analyzed using Review Manager 5.3.5 (The Nordic Cochrane Centre, The Cochrane Collaboration, Copenhagen). Meta-analysis indicated a significant association between DASS and arteriovenous fistula (AVF) or arteriovenous graft (AVG) procedures. Radiocephalic AVF (RC-AVF) and distal endovascular AVF procedures were favored. Various interventions addressed venous narrowing, including simple plication and loop interposition. The Modified by Inserted Latex Link for Endovascular Repair (MILLER) technique, DRIL, Extension Technique, and Proximalization of Arterial Inflow (PAI) were assessed for arterial bypass graft and blood supply preservation. This study underscores the importance of individualized strategies in preventing DASS during hemodialysis access surgery. Prophylactic measures, such as the extension technique, show promise, while DRIL remains effective in treatment. Ongoing research is imperative for optimizing outcomes in this complex patient population.

## Introduction and background

Chronic kidney disease and its advanced stage, end-stage renal disease (ESRD), often necessitate dialysis as a life-sustaining therapy. The cornerstone of efficient dialysis lies not only in the successful creation of vascular access but also in the complex management of its associated complications. Autogenous arteriovenous fistulas (AVFs) and arteriovenous grafts (AVGs) have revolutionized vascular access, offering advantages such as extended patency rates [1]. However, as with any medical intervention, challenges persist, and one such complication that demands careful consideration is Dialysis Access-Associated Steal Syndrome (DASS).

DASS, characterized by the redirection of arterial blood flow away from the extremities, poses a significant threat to patients undergoing dialysis, particularly affecting 10% of diabetic and frail geriatric populations undergoing AV-access surgery [2]. This syndrome not only jeopardizes the success of the dialysis procedure but can also lead to hospitalization, emphasizing the critical need for preventive strategies.

In response to this clinical challenge, various surgical interventions have been introduced to mitigate the risk of DASS. Among them, distal revascularization with interval ligation (DRIL) and the extension technique stand out as promising approaches [3]. The extension technique, in particular, deviates from traditional AVF placement by strategically selecting a more distal site, such as the radial or ulnar artery, thereby reducing the risk of DASS [4-6].

This systematic review aims to delve into the outcomes of prophylactic surgical procedures when coupled with primary AV access surgery, seeking not only to address the immediate concern of DASS but also to contribute valuable insights into preventive strategies tailored for susceptible patient populations. Against the backdrop of the broader challenges of managing ESRD, this study offers an exploration of interventions that not only optimize dialysis efficiency but also enhance the overall well-being of patients navigating the complexities of chronic kidney disease.

## Review

Methods

Literature Search

We systematically searched PubMed, Cochrane Library, EMBASE, and Web of Science databases from their inception until 2022. Medical Subject Headings (MeSH) terms included chronic renal failure, dialysis, vascular access, repair, arteriovenous fistula, arteriovenous graft, aneurysm, cardiac failure, sequel, thrombosis, steal syndrome, and complications. Manual searches of references in recognized publications and reviews were also conducted. The language of publication was restricted to English.

Inclusion and Exclusion Criteria

The inclusion criteria comprised randomized clinical trials and observational studies involving non-pediatric hemodialysis patients, reporting at least one relevant outcome. Exclusion criteria included case series, editorials, comments, reviews, non-original outcome data, pediatric populations, pregnant women, and duplicate publications. The primary outcome was securing patency (primary, assisted, or secondary), while secondary outcomes included cardiac failure, developing an aneurysm, ischemia, or post-surgical complications.

Study Selection

**Figure 1:**
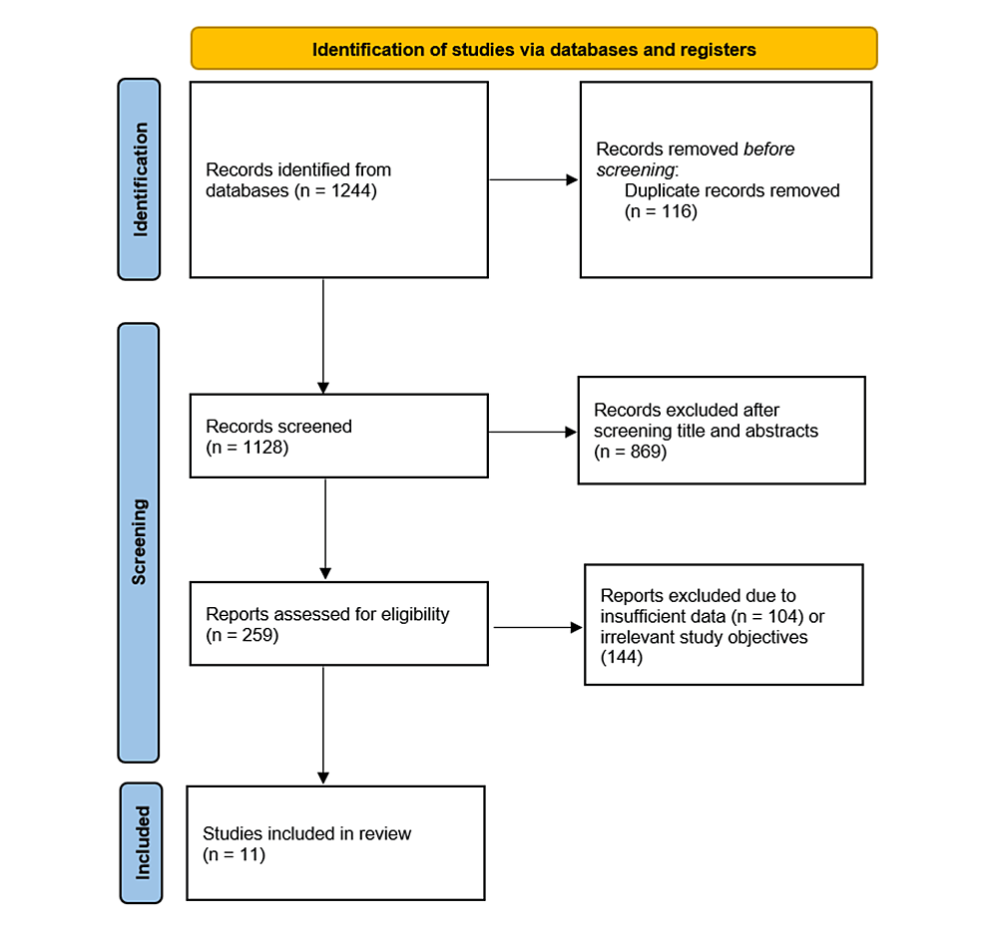
PRISMA 2020 flow diagram

A total of 1244 works were identified in the initial search. After discarding 116 duplicates, 869 records were excluded based on irrelevant titles or abstracts. A full-text assessment was conducted for the remaining articles, resulting in the exclusion of 248 studies that did not meet the inclusion criteria. Ultimately, 11 studies were included (Figure 1) [7-17].

Data Extraction

Two authors independently extracted key parameters, including study design, trial duration, publication date, participant demographics, and reported outcomes. Data were retrieved for multiple groups when applicable.

Data Analysis

**Figure 2:**
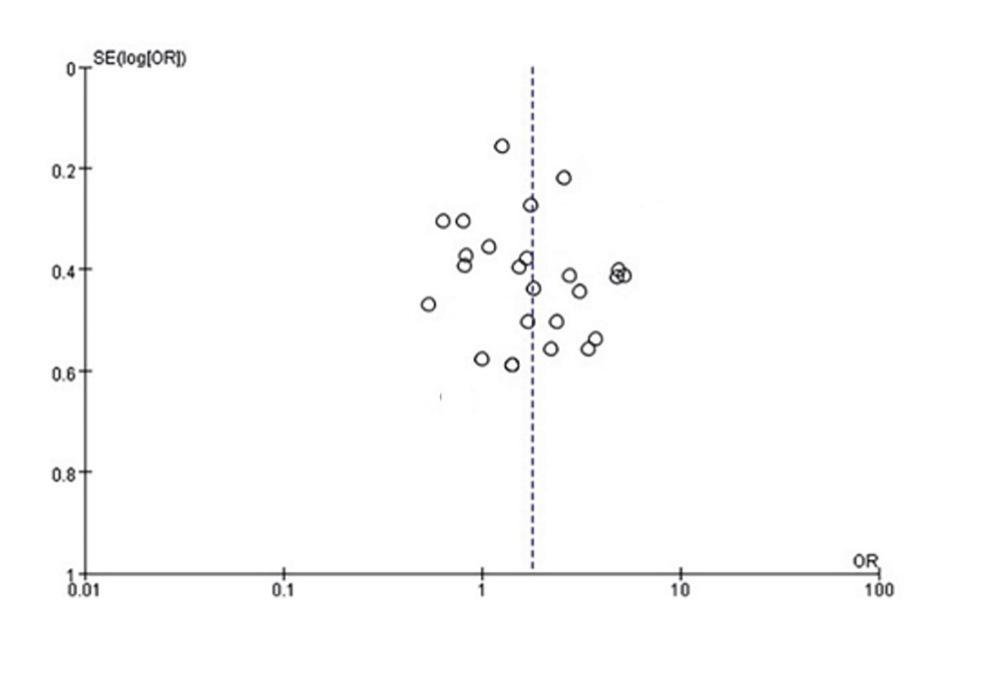
Funnel plot illustrating the 24-month primary patency rates

Review Manager 5.3.5 was used for data analysis, with statistical significance set at an alpha level of 0.05. The effect size was calculated by summing the mean percentage point improvement from the initial condition and standard deviations. Subgroup analyses were conducted based on investigation time and the development of steal syndrome post-hemodialysis. Quantitative and qualitative publication bias assessments were performed using Egger’s linear regression test, Begg’s correlation rank, and a funnel plot. A sensitivity analysis was also conducted. A cumulative Z-curve graph was generated using the O’Brien-Fleming B-spending function. High heterogeneity for the 12-month primary patency necessitated a sensitivity analysis, which did not impact all DASS and CAS results (Figure 2).

Risk of Bias Assessment

**Figure 3:**
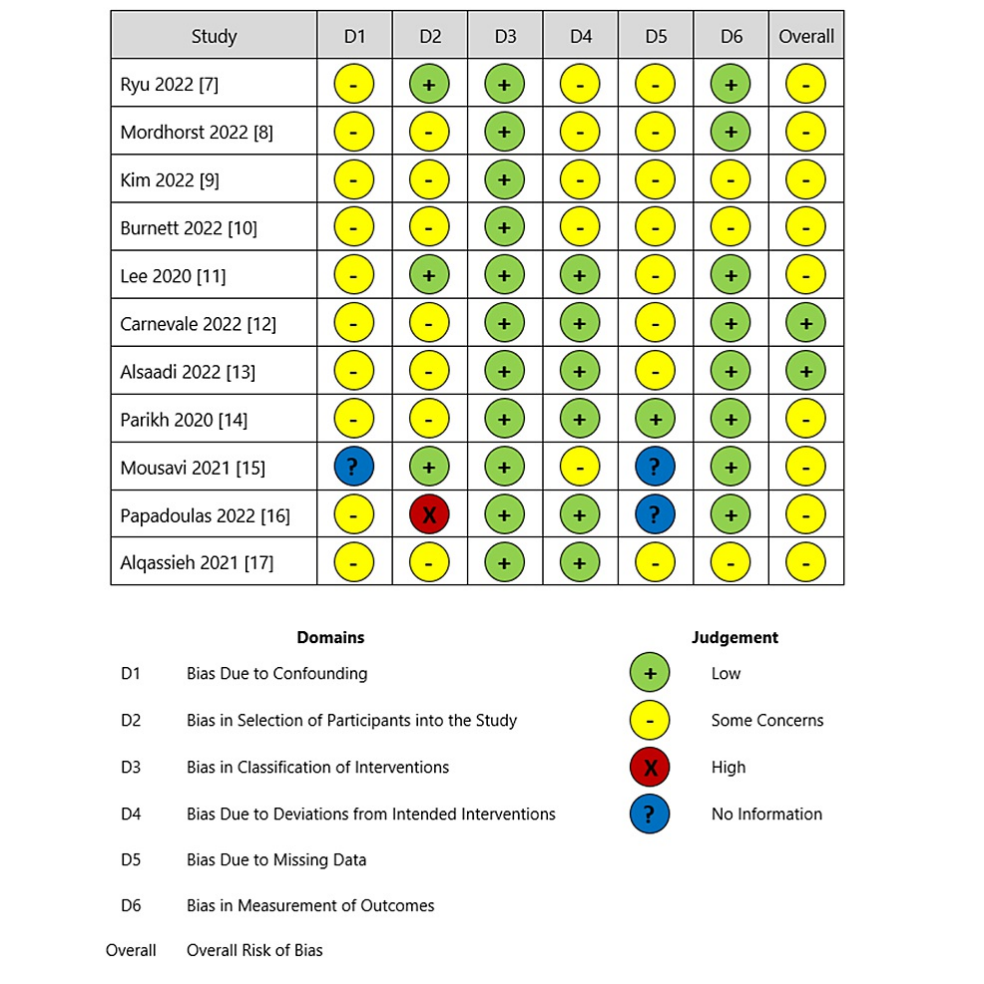
Summary of risk of bias assessment Ryu et al. [7], Mordhorst et al. [8], Kim et al. [9], Burnett et al. [10], Lee et al. [11], Carnevale et al. [12], Alsaadi [13], Parikh et al. [14], Mousavi et al. [15], Papadoulas et al. [16], and Alqassieh et al. [17].

The risk of bias assessment for this systematic review and meta-analysis was conducted to ensure the methodological robustness of the included studies. A thorough literature search was carried out across multiple databases, and inclusion and exclusion criteria were applied to select studies relevant to the research question. Two independent authors performed data extraction, enhancing the reliability of the assessed information. The risk of bias within each study was systematically evaluated, taking into account bias due to confounding, selection of participants, classification of interventions, deviations from intended interventions, and missing data (Figure 3). Furthermore, publication bias was assessed using Egger’s linear regression test, Begg’s correlation rank, and a funnel plot.

Results

Association Between CAS or DASS and AVF or AVG Procedures

Our meta-analysis revealed a significant association between the development of CAS or DASS following AVF or AVG procedures (Table 1). The underlying mechanism involves stenosis-based altered flow, leading to preferential flows through the AVF or AVG, bypassing downstream tissues and inducing an ischemic limb, predisposing patients to DASS.

**Table 1:** Summary of studies BC-AVF: brachiocephalic arteriovenous fistula, RC-AVF: radiocephalic arteriovenous fistula, CAS: cephalic arch stenosis, SS: steal syndrome, NA: not applicable, AVF: arteriovenous fistula, ICC: anastomosis diameter and flow ratio, PAD: peripheral arterial disease, PAI: proximalization of arterial inflow, CABG: coronary artery bypass graft, DASS: dialysis access-associated steal syndrome, HF: heart failure.

Study	Design	Participants	Observation (months)	Results	Conclusion	Other Variables
Ryu and Lee [7]	Retrospective	194 patients from 2017 to 2019 (2 groups; n = 97 each)	~19	Primary patency: BC-AVF accesses > RC-AVF accesses; patency duration: BC-AVF≈ BC-AVF; complications: BC-AVF: 3 cephalic arch stenosis (CAS); mortality rate: BC-AVF group > RC-AVF group.	BC-AVF is comparable to > RC-AVF	NA
Mordhorst et al. [8]	Retrospective	171 RC-AVF, and 137 BC-AVF	~17	Complications: BC-AVF: 11 SS; RC-AVF: 7 SS	RC-AVF achieves the greatest maturation rates and secondary patency	Endo AVF EverlinQ/WavelinQ
Kim et al. [9]	Retrospective	94 octogenarian patients; 66 RC-AVF, and 28 BC-AVF	18	Unassisted maturation rate: BC-AVF accesses > RC-AVF accesses	BC AVF fits octogenarians more than RC-AVF	NA
Burnett et al. [10]	Retrospective	151 RC-AVF, and 36 BC-AVF	18	CAS: BC-AVF accesses > RC-AVF accesses	VF flow rate tends to predispose CAS with BC-AVF	NA
Lee et al. [11]	Retrospective	Registry of vascular quality initiative dialysis access (9 years)	12	Comorbidities: DASS favors diabetic white females with upper arm prosthetic grafts placed in larger target vessels. Secondary patency decreased at 12 months with banding.	The open revision or endovascular	NA
Carnevale et al. [12]	Retrospective	48 patients	17	Comorbidities: diabetes, hypertension, CABG, PAD, and hyperlipidemia with high flow DASS were often banded, whereas those with low flow were typically updated with PAI.	PAI	DASS
Alsaadi [13]	Prospective	49 patients	21	Steal and non-steal anastomosis diameter and flow ratio are proportionally related. Patients with a 1.05 anastomosis ratio or flow ratio of 0.98 are more likely to develop DASS.	AVF, ICC	NA
Parikh et al. [14]	Retrospective	77 patients	18	The mean taper survival was 20.2 months. Pre- and post-taper reduction access flows were 574,315 ml/min and 929,352 ml/min. Six months after tapering, no individuals experienced steal syndrome.	A 6-month reduction in dialysis graft arterial anastomotic taper did not cause steal syndrome. In patients with flow measurements, taper reduction virtually doubled access flow, a critical predictor of access function.	Hemodialysis steal syndrome
Mousavi et al. [15]	Cross-sectional	294 patients	~20	BC-AVF 20 relates to ischemic DASS	The largest prevalence of steal syndrome was seen in the brachiocephalic approach, which is consistent with earlier investigations.	DASS
Papadoulas et al. [16]	Mixture	120	~17	DASS management	In the absence of ischemia, these approaches are utilized to reduce access flow and protect the heart. Patient history, focused clinical exam, color duplex ultrasonography, pulse oximetry, and angiography are necessary.	DASS
Alqassieh et al. [17]	Retrospective	13 patients	~22	The MILLER method is a successful therapy for DASS and PHT/HF connected to dialysis access. Maintaining the graft or stent in place after the index operation may require further interventions.	DASS, pulmonary hypertension, and heart failure	NA

Preferences for AVF Types and Endovascular Approaches

**Figure 4:**
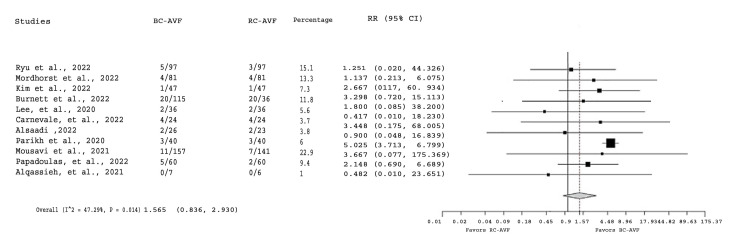
Forest plot depicting the association between steal syndrome and hemodialysis vascular outcomes The studies included in this analysis are as follows: Ryu et al. [7], Mordhorst et al. [8], Kim et al. [9], Burnett et al. [10], Lee et al. [11], Carnevale et al. [12], Alsaadi [13], Parikh et al. [14], Mousavi et al. [15], Papadoulas et al. [16], and Alqassieh et al. [17].

Studies included in the meta-analysis favored the use of RC-AVF, preferably distal rather than proximal, over brachiocephalic AVF (BC-AVF). Additionally, endovascular AVF procedures demonstrated promising results (Figure 4).

Management Strategies for Venous Narrowing

When dealing with narrowing limited to a short vein segment, two commonly employed procedures include simple plication, a straightforward and efficient intervention, and loop interposition, which effectively elongates the short vein segment, offering an alternative solution for specific cases.

In instances of narrowing extending to a long vein segment, two distinct procedures are often utilized. Banding is one such method, allowing for the adjustment of the synthetic cuff diameter and providing a tailored and effective solution for addressing long vein segments.

Interventions for Arterial Bypass Graft and Blood Supply Preservation

The MILLER technique, enhanced by the insertion of a latex link for endovascular repair, enables precise adjustment of vein diameter using a balloon but requires concurrent balloon angioplasty.

Two notable approaches include DRIL, utilizing an arterial bypass graft to restore flow, though requiring a surgical bypass graft, and the Extension Technique, which preserves half of the blood supply to the hand, contingent on specific anatomical conditions. Another method is the PAI, which involves moving the fistula inflow to a proximal artery. It is effective but requires a graft and surgical expertise.

The choice of interventions is patient-specific. DRIL is often applied when the native artery below the fistula is occluded or stenosed, whereas extension, or PAI, is considered when the fistula supplies insufficient blood flow to the hand, underscoring the tailored nature of these interventions based on individual patient needs.

Discussion

Steal syndrome, characterized by the diversion of blood flow from distal tissues to the fistula or graft, poses a significant risk of ischemia in the extremities. This phenomenon diminishes perfusion and oxygenation, resulting in discomfort, ischemic neuropathy, and potential complications such as ulceration and gangrene. The reported incidence of hand ischemia varies, with patients with radiocephalic AVFs (RC-AVFs) and BC-AVFs experiencing 2.3% and 3.6% incidence, respectively [18,19]. The dynamics of access-induced ischemia involve antegrade flow usage and retrograde flow from the native artery distal to the fistula origin [20].

Various surgical approaches address symptomatic hemodialysis-access-induced ischemia with differing success rates. Arteriovenous-access ligation, the least invasive procedure, becomes necessary when the original access site is no longer viable [21-23]. Tapering grafts or fistula banding offer alternatives, each influencing flow dynamics to mitigate the risk of ischemia [24]. Banding, in particular, closes off the inflow opening, redirecting extremity flows and reducing the likelihood of ischemia [5].

Our analysis revealed that in high-flow DASS patients with comorbidities, banding was a common approach. Conversely, those with low flow often received PAI. By moving the arterial anastomosis more proximally, PAI increases blood pressure and flow to the affected tissue. DRIL, another effective intervention, creates a new arterial inflow while ligating the old inflow, improving blood flow to the affected tissue while preserving the fistula or graft.

Efficient hemodialysis access management is pivotal in reducing the incidence of DASS and heart failure symptoms. While open surgical methods may address both DASS and high-output heart failure [25,26], the potential loss of dialysis access remains a drawback. Surgical interventions, including distal inflow or proximalization of arterial inflow using methods like DRIL, should be approached cautiously in high-risk patients. The delicate balance between preserving dialysis access and managing complications underscores the need for individualized treatment strategies.

## Conclusions

In conclusion, our study adds valuable insights to the existing medical literature on preventive strategies for DASS in high-risk patients undergoing hemodialysis access surgery. By reviewing and analyzing the available data, we reinforce the significant link between DASS and specific procedures while emphasizing the importance of tailored interventions. Our findings support the effectiveness of certain techniques, such as the extension method, in preventing DASS and highlight the ongoing need for personalized approaches to optimizing outcomes for patients undergoing hemodialysis.
